# High-level personal trainer perspective for industry practice and development in Serbia: a qualitative descriptive study

**DOI:** 10.3389/fspor.2025.1549979

**Published:** 2025-02-14

**Authors:** Ivan Curovic, David Grecic

**Affiliations:** ^1^ Institute of Coaching and Performance, University of Central Lancashire, Preston, United Kingdom; ^2^ Centre for Applied Sport, Physical Activity and Performance, University of Central Lancashire, Preston, United Kingdom

**Keywords:** exercise, obesity, sedentary, behaviour change, motivation, physical literacy, educational background, professional experiences

## Abstract

Personal trainers (PTs) play a pivotal role in promoting positive gym experiences, implementing effective weight loss strategies, and influencing healthier lifestyle choices. In Serbia, however, the absence of national accrediting bodies for standardised qualifications has led to a lack of formal recognition of exercise professionals. The views of personal training clients regarding their training practices and experiences have been explored previously. Building on this, the current study investigated the perspectives of high-level Serbian PTs, examining their professional developmental journeys, educational background, coaching philosophies, professional needs and experiences. The aim was to provide insights for educational institutions and other practitioners to adopt successful approaches that inspire lifelong exercise and promote positive lifestyle changes for increasingly sedentary populations. Using a snowball sampling method, we interviewed 12 high-level PTs, selected for their exceptional practice as recognised by their peers. The findings suggest a need for a shift from traditional, physical performance-focused approaches toward a more holistic model that incorporates psychosocial support and a care for the whole person, fostering an appreciation for the concept of physical literacy. Additionally, the study identifies critical gaps in PTs education and training, particularly concerning the lack of focus on behavior change models and inquiry-based skills essential for their future self-directed learning. These areas were highlighted as vital for continued professional development after formal qualifications are obtained. In conclusion, this research underlines the need for holistic training approaches to improve clients' physical and health outcomes. The study offers guidance to shape industry standards, foster accreditation systems, and start the debate on how best to continually support PTs during their careers. Such action is essential if the evolving personal training profession in Serbia is to be equipped with the tools needed to promote long-term client engagement and achieve the health benefits for the wider Serbian population.

## 1 Introduction

Modern humans are leading increasingly sedentary lives that have negative health repercussions on a global scale (1). These inactive lifestyles associated with the modern world we live in are not only damaging for physical functioning (2) and metabolic health (3), but they also negatively affect mental health and wellbeing (4, 5), thereby presenting a daunting perspective for future generations. This “quiet epidemic” requires targeted interventions and strategies to prevent its devastating consequences for different population categories, including older adults (6), young adults (7) and children (8).

The World Health Organization has continuously called for action to develop and implement effective strategies to reduce global deaths and diseases by encouraging physical activities for life (9, 10). Physical activity levels may be obtained via non-exercise daily activities (e.g., walking to work or climbing the stairs) (11), or by structured exercise forms that could take place outside or indoors (i.e., gym spaces). Exercise is a type of physical activity specifically aimed at improving health or physical capacity. As such, it has attracted immense research interest over the last decade with its effects toward multiple health outcomes (12–15). Consequently, it has been shown that any form of exercise has potential to target and improve the functioning of multiple organs and tissues across the body (13, 16, 17). Therefore, motivating people to engage in physical activity is one of the primary messages highlighted by preventive health interventions and promotions (18–22).

Personal fitness trainers (PT) are among the first to translate medical advice into focused exercise interventions (23). This profession requires a holistic pool of knowledge, including medical-biological, social-humanitarian and psychological disciplines (24, 25). Furthermore, PTs play a vital role in promoting healthy lifestyles by encouraging regular physical activity engagement (26). Importantly, this influence can extend beyond the individual client, creating a “chain reaction” where those inspired to adopt a healthier lifestyle and engage in exercise become sources of social support for others in their surrounding (27–29). This underscores potential broader societal impact of an inspiring PT. Therefore, in addition to possessing a deep understanding of exercise sciences, trainers should provide a comprehensive socio-emotional support to their clients, fostering positive attitudes not only with the fitness routines, but also with the behaviour outside of the gym spaces (30).

While the educational opportunities and degrees in personal fitness training profession seem to be well established in western countries such as the United States and United Kingdom (31, 32), Serbia does not have standardised requirements (30) or an accrediting body apart from its university courses (33). Currently, there are around 1,000 fitness centres in Serbia (34), yet there is no mandatory certification required to work as a PT. This lack of regulation has resulted in a disjointed market, with independent organisations providing their own unique courses and awarding their own or external certifications [see (35–38):]. That said, there is a recognised need for improved education for coaches by relevant institutions and a stronger public message on the importance of exercise (34). In the absence of a body of research on Serbian PTs and their work, we have previously investigated clients’ perspectives from three elite Serbian training centres which established the qualities that characterised a successful PT and revealed working practices that were more and less appreciated (30). The responses of 148 participants suggested that the most desired PT qualities were professionalism, social competency and motivational support to foster lasting lifestyle modifications, while the greatest challenge to clients was the motivation to apply behaviour and dietary changes (30). The next step in our investigations is a qualitative study aimed at providing insights into the PTs own philosophy and behaviours, as well as strategies that could enhance personal fitness training practice in Serbia, where the absence of an accrediting body leaves the profession without standardised requirements. This study, therefore, explored the perspectives of 12 high-level PTs operating at the most respected facilities in the country. Through the examination of their methods, philosophies and experiences, this research aims to inform the profession and offer practical recommendations that could benefit both practitioners and the Serbian public. Thus, our investigation is guided by the research question: “What do Serbian high-level PTs do and what has contributed to their professional development?” By investigating this topic, it is hoped it could provide another step forward in advancing the understanding of how this important profession can contribute to the improvement of public health, addressing the obesity epidemic in Serbia (39) and beyond.

## 2 Methodology

### 2.1 Study design

This study applied a qualitative descriptive design guided by an interpretative philosophy with the goal to investigate practices and developmental pathways of high-level PTs in Serbia. The aim was to uncover rich insights into their professional learning and coaching experiences, philosophies and methods of work. This information intended to inform educational institutions and enhance the preparation of future PTs by identifying key areas for development. Such interpretivist research allows for the exploration of the complexity of the social world through the investigation of individual lived experiences (40), and sense-making of these experiences (41). It is an approach that has increasingly been applied to studies in the sports and fitness training professions (42, 43). Furthermore, Smith and Sparkes (44) explain that the interpretivist paradigm allows the researcher to gain deep insights into individual issues within social worlds, constructed by interests, emotions, and values. Resultantly, this paradigm was used within the study, with data collected through in depth semi-structured interviews with 12 high level PTs in Serbia.

### 2.2 Participants and sampling

The study involved the recruitment of 12 high-level PTs. The term “high level” was selected due to the absence of an industry recognised definition of expertise (Table 1). Modified criteria were therefore selected to ensure a high level of participants in line with similar criteria applied in related disciplines (45, 46). Specifically, these criteria were that each PT had a minimum of ten years of professional experience, had demonstrable success in the field such as maintaining a large client base, had achieved significant results with clients (e.g., measurable weight loss), and had demonstrated an invested interest in their clients' wellbeing and progress as perceived by their peers. Participants were identified through a snowball sampling method (47, 48), informed by peer recommendations (i.e., gym managers and colleagues) selected from reputable personal fitness training centres. Contacts were initially provided by gym managers, and subsequent participants were identified through referrals. Recommendations ensured the selection of trainers recognised for their expertise and reputation within the Serbian fitness community. The study was approved by the University of Central Lancashire Institutional Ethics Panel for Behaviour, Art, Health and Social Sciences board (ref. BAHSS2 0296).

**Table 1:** Demographic information of the interviewed personal trainers (mean age: 36.08 ± 3.42 years; mean experience: 13.92 ± 2.47 years).

Personal trainer	Age	Qualification (degree/certification)	Working experience (Years)	Client Population
PT1	35	Undergraduate	14	Recreational, clinical, athletic
PT2	36	Master's degree	15	Recreational, clinical, elite athletic
PT3	36	Master's degree	15	Recreational, clinical, elite athletic
PT4	37	Master's degree	15	Recreational, clinical, elite athletic
PT5	37	Undergraduate	15	Recreational, clinical, rehab
PT6	40	Undergraduate	17	Recreational, elite athletic, rehab
PT7	43	Master's degree	10	Recreational, athletic
PT8	35	Master's degree	15	Recreational, clinical, rehab
PT9	29	Undergraduate	10	Recreational, clinical
PT10	37	Master's degree	16	Recreational, clinical, athletic
PT11	35	Master's degree	15	Recreational, clinical, rehab
PT12	33	Undergraduate	10	Recreational, clinical

### 2.3 Data collection

All participants were males (*n* = 12). Prior to the interview, PTs were emailed an information sheet, and informed consent was obtained. Semi-structured interviews were designed to explore the trainers' philosophies, client relationships, professional objectives, practical methods, and factors influencing their career directions. The interview guide was structured into five thematic sections:

1. Philosophy (3 main questions)
2. Trainer-Client Relationships (4 main questions)
3. Objectives and Intentions (5 main questions)
4. Practice Methods (3 main questions)
5. Factors of Influence (4 main questions)

Each section contained sub-questions to probe further where detailed insights were not initially obtained. Questions were carefully crafted to gain reflective responses, providing a thorough understanding of the trainers' approaches and developmental pathways. For example, questions relating to philosophy included, “*What is your overall coaching philosophy*?” “*Where did this come from?*” and “*Has this evolved throughout your career?*” Examples of follow-up probes used were, “*How?*” “*What does that look like in practice?*” “*Can you give me an example?*” and “*Why did this change?*”. Interviews were conducted in a quiet office setting by one of the authors (IC), a certified PT, ensuring a shared professional language and comprehensive rapport with participants. Interviews were audio-recorded for accuracy and later transcribed and translated from Serbian to English by the interviewer. The mean duration of interviews was 37 min, ranging from 20 to 60 min. All the interviewers were scheduled based on participants' availability (full schedule is available upon request).

### 2.4 Data analysis

For this study, and in line with its pragmatic focus and interpretivist nature, reflexive thematic analysis (RTA) was utilised with all six stages of the process guided by Braun and Clarke's work (49–51). RTA is a widely used method in qualitative sport and exercise research with its point of difference to other thematic analysis methods being that the researcher's position and contribution is necessary, unavoidable, and an integral ingredient of the process (49–51). In the present study, the first author is a qualified and experienced PT and an university lecturer. He is a Serbian national and has worked in the Serbian fitness industry for over 15 years. The second author, being of Serbian heritage, has a high understanding of the Serbian sport, exercise and physical education environment. He is also a highly experienced researcher in qualitative methods. In RTA, such subjectivity evident by both researchers are tools to be valued and drawn upon rather than to be removed or avoided (49, 50).

Braun and Clarke's (49–51) six-stage process for RTA first involves a refamiliarisation with the data, followed by the generation of initial codes. Here, the second author (DG) worked systematically through each response, identifying aspects that were interesting, and potentially informative in developing themes. The third stage involved the early generation of themes with the coded data reviewed and organised. Eventually, two storybook themes from six major themes and sixteen sub-themes were created. Codes were combined based on the similarity of concept, language and/or perceived significance, and these combinations of codes were interpreted to form meaning for the other author (IC). The next stage involved a recursive review of the final storybook themes in relation to the coded data and entire dataset (51). Here, sub-themes and themes were refined and modified, and sense-checking was undertaken by the first author along with external PTs who had agreed to support the study. Finally, the resultant themes were named, defined and written up within the Results section (presented below in Table 2).

**Table 2:** Thematic analysis of personal trainers’ industry experiences and insights.

Storybook theme	Major theme	Sub theme
Becoming an effective personal trainer	We do not know everything	University foundation
		Gaps in knowledge
		Sharing experiences
	In-practice learning	Self-driven and self-directed
		Significant others
		Being adaptable and creative
	Challenges to the profession	The resource envelope
		Others poor practice
		Diminished trust
Being a personal trainer	It is not just sets and reps	Holistic focus
		The power of motivation
		Nested goals
	Getting to know the person	Building trust
		What clients want vs. what they need
	Desired outcomes and impact measurement	Healthy lifestyles
		Performance goals drive practice
		Ownership of effort

### 2.5 Trustworthiness

Smith and McGannon (52) recognise that rigour has been “largely been described as a marker of excellence sought through method.” Aligning to the Big Q interpretivist positioning of RTA (53), and to maintain philosophical alignment, a relativist approach to trustworthiness was adopted, and internal markers of quality were utilised based on Tracy's “Big Tent approach” (54), thereby maximising the interpretation, backgrounds and lived experiences of the researchers and the reader. Specifically for this study, we direct the reader's attention towards how the data presented not only “tells a story” but is also perceived as providing a substantive contribution to a worthy topic, and offers rich and fulsome data that are transparently presented in the *Results* section (52).

## 3 Results

Table 1 provides demographic information relating to the cohort participants represented. Following the interviews, participant data was anonymised with each participant being randomly assigned a designation and number (PT 1–12).

Initially, 284 raw data codes were identified. Data clusters were then developed and grouped together to reflect the meaning associated to them by the researchers. This led to the development of 16 lower order sub-themes, 6 mid-order major themes, and, finally, two higher order storybook themes (Table 2).

### 3.1 Storybook 1: becoming an effective personal trainer

Storybook theme of “*Becoming an effective PT*” referred to the PT's personal and professional development. It describes the self-perceived gaps in educational training and the strategies they have employed to fill them. This theme is constructed from the major themes of “*We don’t know everything*,” “*In-practice learning*,” and “*Challenges to the profession*.”

#### 3.1.1 We do not know everything

The overarching messaging from within this theme was that university education and courses had provided the PTs with notable foundations that needed to be built upon in order to be an effective PT. For example, PT1 described the input of his university as, “*They certainly directed us and required a broad base of theoretical knowledge. In general, I think the university gave us a good foundation.. but it didn’t give us a practical model*.” Despite recognising the role university degrees had played in providing a useful knowledge base, many highlighted that this was only the start of their learning journey, and it was they, through individual effort and experience that had been digging into the more important areas of development. As PT9 stated, “*My formal education gave me a foundation to work as a personal trainer. It provided me with the basic knowledge to start my career. However, I would say that a significant part of my expertise came from other factors like self-learning and mentorship. Over time, I’ve gained valuable insights through practical experience and by learning from more experienced trainers, especially my mentor. These hands-on experiences and mentorship opportunity to learn directly from one of the biggest experts in this profession have shaped my current approach to training*.” Indeed, when the PTs looked back on their time at university and the content and experiences they had covered, they highlighted large areas of missing knowledge and important topics which they had been totally unaware of as well as the lack of resources for the future independent learning journey. PT3 provided more detail explaining, “*Although my university helped me gain the factual theoretical knowledge from subjects such as anatomy or physiology, these subjects were separated in between the learning years and they lacked pragmatic approach in preparing us for the work and deeper connection to the actual training practices*.” He elucidated saying that “*Only when I graduated, I started to actually connect the dots, learn, study, explore especially in regard to the weight loss principles and psychosocial factors that influence the work with real people. Perhaps the most notable flaw was that we missed the inquiring skills that direct self-learning further which made the future learning process harder*.” PT11 supported this point but did acknowledge the initial value of his university degree:

Regarding my formal education, I do believe it provided me with a solid foundation of knowledge. The academic background I gained is valuable, and clients recognize and appreciate it. However, to keep growing as a personal trainer, I had to go beyond what I learned in school. Continuous self-learning and real-world experience have contributed more to my expertise than my formal education alone. I had to dig deeper, learn from experience, and expand my knowledge to become truly effective in my work.

This reflection was shared by most PT's interviewed, with PT5 reinforcing the above points describing his own experience as:

I did not acquire the knowledge at university to become a successful trainer, but rather just a foundation for further development in the profession. I started independent practice in my third year of studies at a fitness club that I found on my own (not connected to university practice), and that's where it all began. I believe that after graduation, without additional practical experience, a student is not equipped to be a successful personal trainer. In fact, it was during later training courses that I realised how much I hadn't learned at university despite spending countless hours on studying.

As the PTs developed their roles and shared their experiences with their peers, they reinforced the opinion that valuable topics they had later learnt informally or non-formally had been missing from their earlier formal university degree. PT4 placed relative values on where his knowledge had come from when he looked back upon his whole career. He described his development as being dependent on a “*combination of good mentorship (other experts taking time to help me) and live practice …So, knowledge from University is 35% and other 65% is my own ‘in person’ practice*.” This reflection from leads nicely into the next major theme that was constructed from the PT data, that of *In-practice learning*.

#### 3.1.2 In-practice learning

Expanding upon the points raised above, this major theme provided greater detail into how the PTs had sought to plug the gaps they recognised in their own and others' practice. A number of PT's lamented the absence of clear professional development routes linked to overarching associations that would provide, monitor and accredit learning that was fit for purpose. For example, PT2 linked this point back to the university preparation revealing:

I don't believe that the university provided me with the knowledge necessary to work as a fitness trainer, but thanks to the university, I was able to more quickly connect the knowledge I picked up along the way. Compared to other courses in Serbia that produced a lot of PTs who do not know much about this profession, the university degree serves a good basic knowledge because it requires a lot of learning.

In order to fill the perceived gap in provision, many PTs explained how they had taken responsibility themselves in order to address their own weaknesses. They described many self-directed strategies and behaviours that they had adopted in order to learn more about new areas of interest. For example, PT3 stated, “*I cannot say that my expertise in personal training came from my university. It came as a result from my obsessive mindset to improve people's lives and learn everything I could that encompasses this profession like nutrition, supplementation, exercise sciences*.”

The PTs also noted the importance of significant others in their professional journey and how they had utilised role models, communities of practice and more experienced mentors to develop themselves further. What was common amongst this group was the thirst for knowledge and the drive to continuously improve themselves and the service they could provide their clients. For example, PT5 declared that “*While my formal education provided a foundation in exercise science and basic coaching principles, I would say that only around 10% of what I learned in school has been truly useful in my career as a personal trainer. The majority of my expertise has come from self-learning, hands-on experience with clients, and mentorship from experienced trainers and online resources that I trust*.”

A key and common factor appeared to be the use of peers and significant others (more experienced coaches, mentors, friends) to help them on their developmental journey. As PT12 explained, “*A major part of my development came from learning directly from experienced colleagues, who were exceptional in their work. Their mentorship was crucial in helping me understand the nuances of personal training that weren’t covered in my university curriculum*.” PT4 described the community of practice he had developed with his peers, “*I had great luck in my life to work with the colleagues from my university, and those are long time great friends…I respect the fun, the laughs that we have together. It's something I think it's rare, but I'm quite grateful to my life that I have those kind of colleagues. And of course, when they can teach me something new, I am more than happy to learn*.”

In respect to the PTs' own thought processes that drove their self-development, many described a critical thinking approach when coming across new information and a creativity in how they attempted to apply and test these ideas and theories. PT1 illustrated this by describing his training methods, “*I am trying to be innovative. I thoroughly enjoy being creative with this goal in mind*.” He then clarified his approach. “*I try to constantly improve my knowledge about training, so yes, I have changed and I am changing a lot, along with the sports science findings and guidelines. What was considered good before is not considered so good now, so you need to critically approach to training individuals*.” Indeed, PT10 provided specific examples of how his personal training service had evolved by aspiring to learn about and deliver new concepts to his clients:

I've incorporated additional elements into my coaching, particularly focusing on mental resilience, lifestyle habits, and overall well-being. I now spend more time working with clients on goal setting, stress management, and fostering a positive mindset toward fitness. I've also added nutritional guidance and recovery strategies to help clients make more sustainable changes in their lives. This shift to a more holistic approach has made a big difference in the results my clients achieve and has helped them maintain progress long after our sessions.

#### 3.1.3 Challenges to the profession

This major theme within *Becoming an effective PT* presents a number of issues which the PTs perceived could, and in some instances had, had a negative impact upon their development. An obvious element highlighted by many was the lack of resources afforded to the PTs, whether this was related to time, ease of access to learning materials, the equipment and facilities to practice and upskill, and the relevance and quality of professional development opportunities. For example, PT4 highlighted the impact of financial concerns by explaining, “*financial instability is something that influences your quality of coaching because you must work a lot and this takes a lot of your time and consequently negatively influences your focus and probably quality of work. Even though you love your job, sometimes it sucks up your energy and time. That's probably one thing with this surrounding that has negative side for our profession*.” Indeed, in an effort to plug the gaps in knowledge the PTs declared one of their biggest challenges being, “*the pressure to stay updated with the latest research and industry trends. This requires continuous learning, which takes time away from coaching and can sometimes feel overwhelming, especially when balancing it with the operational side of running a coaching business—like marketing, client acquisition, and administrative tasks. This added workload can sometimes limit the time and focus I can dedicate to personalising coaching plans for individual clients or introducing new ideas*”—PT10. In fact, PT10 provided a very detailed outline of exactly what PTs needed to spend time learning about to improve their practice:

Topics like behavior change strategies, weight loss techniques, and practical coaching skills were not adequately covered during my formal education, so I had to seek out that knowledge independently. This was a significant gap in my formal training. I had to learn these concepts on my own through self-study and real-world experience. In my opinion, behavior change strategies, along with marketing, self-management, and client retention skills, should be a larger focus in personal training education, as they are essential for helping clients make long-term changes.

Finally, what was happening within the PT's world and immediate environment was discussed as a limiting factor for the profession. PTs recognised they were stereotypically seen as incompetent by the public due to the negative connotations projected onto them due to the poor practice by the majority of PT professionals. PT8 explained why this was occurring when he stated that “*many colleagues decide to pursue this career because of the easy money while they actually do not really know their jobs. For this reason, I believe that this profession is getting undeserved lack of respect*.” PT12, however, was a little more forgiving believing that many PT's “*follow the most basic of approaches due to a fear of the unknown. We should not be so proud to think that we do not need to change. Change is good and rethinking is valuable*.”

### 3.2 Storybook 2: being a personal trainer

The second Storybook theme refers to the PTs' working practices. It links to the first storybook theme, but offers more insight into the evolution of PTs' beliefs and how these influenced new behaviours. It includes the practical things PTs do with clients everyday whilst highlighting how they now prioritise the individual's needs much more, as well as holistic practice, and the psychology of motivation. For example, PT10 stated “*My coaching philosophy is centered around a personalised and holistic approach to fitness and wellness. I tailor programs to meet individual needs, focusing on not just physical fitness but also mental and emotional well-being*.” Additionally, PT10 believed that in order to be a good personal trainer he needed “*to be a good psychologist too—listening, motivating, and supporting clients on a personal level. It's not just about physical training, but also helping clients navigate emotional and lifestyle challenges to achieve lasting change*.” Interestingly, PT7 saw his role as “*helping* [the clients] *not only get fitter, but also build healthier habits through genuine support and connection*.” Of note and interest here, again, is that the PTs felt that all aspects of this praxis had been learnt post-university and outside of their formal training. This Storybook theme is made up of the following three major themes: “*It's not just sets and reps,”* “*Getting to know the person,”* and “*Desired outcomes and impact measurement.”*

#### 3.2.1 It is not just sets and reps

This major theme refers to the PT's reflections on their initial thoughts when they entered the profession and how those had evolved to include a more comprehensive focus on the holistic aspects of human development and not just the scientific mechanics of the “sets and reps”/nuts and bolts of being a PT. In fact, PT4 shared his most recent coaching philosophy as now being, “*not only about the physical approach, but also careful mental preparation and psychological analysis of what you need to do with the client in order to address them as a whole person*.”

The PTs looked back on the effectiveness of their work with clients and championed the power of motivation and their subsequent use of psychological and psycho-social based interventions to help develop this. As an example, PT12 acknowledged this with experience that “*I’ve realised that the most successful transformations happen when clients are self-driven and motivated. While I can provide the tools and guidance, the real progress comes from clients who are willing to put in the effort consistently.*” In this respect his role was to build the “*motivation to commit to building* [the client's] *best selves. My role is to guide, push, and show them the right way, but the drive has to come from within*.” The PTs also explained how their original self-assessment mechanisms and one-dimensional planning had developed into a much more complex process utilising nested goals that targeted different facets of their clients' development, but again with a large focus on developing motivation. PT1 provided a very honest analysis in this regard stating:

I faced issues with the lack of motivation by certain individuals, and this has actually influenced me to come up with the training practice that would stimulate their enthusiasm. Most of the clients know their goals, but sometimes they lack drive to work hard to fulfill these goals. This is when fun sessions and conversation kicks in. You need to motivate your client, otherwise they will not want to engage adequately.

#### 3.2.2 Getting to know the person

This major theme championed how the PTs focused so much of their efforts to learn about their client, their wants and needs, and the drivers and perceived limiters that would bear impact on their work. A large part of the PTs' work therefore revolved around developing and then sustaining/managing a positive relationship with their client. This involved employing conscious strategies to establish rapport and build trust in each other. As PT1 confirmed:

I consciously work to build trust and establish open communication early on. Over time, the relationship deepens naturally as I learn more about the clients' or colleagues' personalities, preferences, and motivations. MS and I incorporate holistic elements like nutrition guidance and mental resilience training to ensure balanced progress. My approach now includes not only physical fitness but also education around healthy habits and recovery, fostering a more sustainable and independent fitness journey for each client.

PT10 reinforced this shared working practice explaining:

I maintain a supportive and personalised relationship with my clients. I strive to build trust by listening to their needs, offering tailored advice, and showing genuine care for their progress, both physically and mentally. My focus is on understanding their individual goals and challenges, helping them not just with workouts but with overall lifestyle improvements. This creates a partnership where clients feel empowered and motivated to take ownership.

Many PTs went a little further and described critical aspects of this relationship and partnership building process. PT9 recognised that the mutual trust he shares with his clients, “*comes from maintaining a professional approach while also developing a close, personal connection. It's not just about training them; it's about creating a safe space where they feel supported*.” PT8 expressed the investment he puts into every client and how this may be what singles out this study's PTs as different from the mainstream practitioners: “*My relationship with clients is built on trust, and that trust is developed through care and closeness. Most clients won’t automatically trust a trainer just because of results or recommendations. I have to earn that trust by showing them that I genuinely care about helping them*.” This point of difference, however, has had to be learnt and is another example of how the PTs' self-development has enabled them to facilitate client gains. As PT7 revealed, “*Early on, I would often approach all clients with similar expectations, assuming that if a particular method or routine worked for one person, it should work for everyone. However, after working with clients who struggled due to factors I hadn’t initially considered—such as stress, mental health, or their lifestyle—it became clear that a one-size-fits-all approach doesn’t work*.” Indeed, this person-centred approach has now been embedded within PTs philosophies and values with PT11 describing his philosophy as, “*all about gradual progress and adapting to each client's unique needs. I believe in making changes that can turn into lifelong habits, so it's essential that I adjust to the client, not the other way around*.”

#### 3.2.3 Desired outcomes and impact measurement

The final major theme in this section reflected how the PTs themselves had changed their outlook on assessing their own effectiveness in their respective roles. For many, this focus has now been projected onto their clients' life away from the gym and within all the one-to-one interactions. PTs spoke about their responsibility to teach, coach and mentor their clients to establish and maintain healthy lifestyles. In particular, PT4 noted how he facilitated this in practice. “*This involves setting realistic, achievable goals, fostering consistency, and promoting long-term habits. I emphasise education, empowering clients to understand their bodies and their progress, and helping them take ownership of their fitness journey.*” PT10 continued to highlight how he too had changed and developed from his early practice stating:

Initially, I focused more on training techniques and achieving short-term goals. However, through experience, I realised the importance of a more holistic approach. Working with a diverse range of clients has shown me that personalized, sustainable solutions work best for long-term success. I've also integrated a greater emphasis on mental resilience, recovery, and education, helping clients take control of their fitness journey and create lasting, healthy habits.

PTs also illustrated how their initial didactic practice had changed beyond a two-way partnership with the client actually leading the process and their performance goals driving the PT's daily practice. Indeed, reflecting the other major themes within this Storybook, the PTs described their new approach to evaluating their work as being the ability to hand over ownership of effort to their client, as long as it was predicated upon a base of fully understanding their client and the application of behaviour change and relationship management protocols. For instance, PT10 explained, “*A significant measure of success is when clients feel confident enough to take control of their fitness journey and maintain healthy habits on their own. I also look at improvements in clients’ motivation, mental resilience, and stress management, ensuring my coaching goes beyond physical fitness*.”

To summarise how this major theme integrates within the Storybook theme, PTs provided examples of what they do now and how they evaluate their positive impact. PT11 described that he sees “*positive changes in [his clients] their attitudes, their performance, and their results. They seem healthier, more mobile, stronger, and they often tell me that they feel more confident in themselves, which is a great indicator that we're on the right track*.” PT9 reinforces this approach stating he focusses on, “*helping clients develop lifelong healthy habits, prevent injury, and maintain long-term physical and mental well-being. I aim to foster independence, so they can confidently manage their fitness and wellness on their own*.” Finally, PT10 praised the new approaches adopted by the PTs explaining that in his own work, “*the shift toward a more holistic, client-centered approach has helped me build stronger relationships and deliver more sustainable results. There are always challenges, but the positive feedback and client retention show that my methods are effective*.”

## 4 Discussion

This study aimed to explore the views of high-level PTs from Serbia about their professional practices and development, examining their methods, philosophies, and experiences. These have provided valuable insights and now offer practical recommendations for both practitioners and educational institutions. The main findings suggest that the personal training industry needs to move away from a traditional performative perspective that focuses solely on physical attributes and instead consider a more holistic approach that embeds psychosocial support and care for the whole person to instigate clients' appreciation of physical activity for life. Many training and educational gaps in preparing PTs for their future role are identified, including a lack of emphasis on behavior change models and inquiry-based skills concerning a self-directed learning approach. Both of these were highlighted as essential areas for continued professional development after PTs had completed their degrees.

In relation to the key findings presented above, the participants underlined the necessity to take ownership of their own professional growth in their successful careers. They frequently described utilising self-directed learning and external resources, including books, online courses, and significant others, to stay informed on the latest advancements in exercise sciences and coaching methods. Of the 12 interviewed coaches, all were university graduates with 7 of them holding a master's level degree. Yet, interestingly, most did not fully appreciate their formal education. This was reflected in responses promoting and prioritising the importance of real-world experience and further learning, even for those with strong academic foundations. This may be due to a traditional, knowledge-focused approach to teaching in Serbia which may lack a holistic interdisciplinary perspective, effectively limiting the practical application of the learned knowledge (55). For example, one PT suggested that “…*subjects (from university) were separated in between the learning years and they lacked pragmatic approach in preparing us for the work and deeper connection to the actual training practices*.” Despite all the interviewed experts clearly being inspired to become lifelong learners, one of the biggest shortcomings of their formal education was, ironically, perceived to be a lack of preparation for continuous learning after graduation. For instance, three PTs highlighted their previous lack of familiarity with inquiry-based skills and resources for the future learning direction, which made it difficult to navigate a learning path on their own. This is in contrast to many developed countries that invest heavily in initiatives aimed at equipping students with the skills to inquire independently, locate reliable sources, and nurture lifelong learning habits (55, 56). In fact, an evidence-based fitness professional will likely benefit more from analytical skills and research-reading techniques than from other forms of learning (56). One may, therefore, consider that our participants have actually overcome many of their environmental constraints with one PT noting that his expertise “*came as a result of my obsessive mindset to improve people's lives and learn everything I could that encompasses this profession including nutrition, supplementation, and exercise sciences*.” This comment is in agreement with findings from a study on PT competencies (57), where exercise practitioners emphasised the need for a broad knowledge base involving medical and exercise sciences as well as psychosocial skills in order to motivate and inspire clients' adherence to exercise (57). Thus, in addition to the familiarisation with exercise strategies for morphological transformations, the profession appears to require an understanding of medical conditions (e.g., diabetes, hypertension), and the capacity to deliver targeted motivational support (27, 30, 57). Therefore, it is not surprising that our participants underlined an educational pathway that goes well beyond what was learned in formal education.

Our investigation revealed a significant gap in PT's previous learning opportunities concerning the psychosocial and pragmatic skills necessary for working with real clients (30, 57, 58). All of the participants agreed about these competencies' importance and stressed that those had had to be picked up through the experiential learning method during their careers. This aligns with findings from a recently published study examining the perspectives of Serbian PT clients, which highlighted the importance of holistic qualities in successful trainers, particularly those encompassing social skills and motivational support (30). Undoubtedly, knowing how to approach and motivate an individual for a positive lifestyle change and long-term exercise adherence is among the most important aspects of a personal training service (30, 58, 59). This is reflected in a multifactorial role of a competent PT which exceeds a solitary physical focus by significantly impacting mental and emotional wellbeing, self-esteem, self-awareness, and social interactions (24, 59–63). Therefore, it would seem critically important for future personal training professionals to nurture holistic approaches to clients, influencing their out-of-gym behaviours and sustained training practice. Indeed, only a happy and socioemotionally satisfied gym-goer is likely to stick to their fitness routines (58, 59, 64). Unfortunately, university courses from many countries such as Serbia still have a strong commitment towards the performative curricula with physical testing as a noted “product” of such approaches (65–69). In contrast, well established contemporary practices emphasise the importance of a whole-person approach, including psychological and social considerations (27, 28, 58, 64, 70, 71). In fact, a biopsychosocial perspective, which suggests that physical activity behavior is influenced by a complex interplay of biological, psychological, and social elements, may be a critical factor in producing exercise practitioners equipped to deliver successful interventions of interest for the public health (59, 72). Nonetheless, cultural context in Serbia presents challenges similar to those faced by other countries in the Balkan Peninsula, which may influence the successful implementation of holistic teaching approaches (55). Traditional views on fitness in the country often prioritise physical performance over psychosocial aspects (34), which may lead to resistance when attempting to shift towards a more pragmatic model. Resultingly, the evolving public perception of fitness and wellness in Serbia might require more time and education to fully embrace such comprehensive strategies to supporting health and wellbeing for Serbian populations.

The primary challenges faced by our PTs included insufficient time to invest in ongoing education and staying updated with advancements in the field. This issue stemmed largely from a high workload driven by financial constraints. Moreover, many expressed frustration over the lack of professional recognition due to the absence of accrediting bodies to validate their qualifications and practice. Many believed that this gap has allowed individuals to enter the profession through various short-term courses, often without adequate education or preparation to work effectively with clients, resulting in poor practice and diminishing public trust in PTs as professionals. For example, one PT mentioned that “…*many colleagues decide to pursue this career because of the easy money while they actually do not really know their jobs. For this reason, I believe that PT profession is getting unjustified lack of respect,”* while the other one suggested that “…*we should not be so proud to think that we do not need to change. Change is good and rethinking is valuable*….” This area may present an opportunity for the educational institutions in Serbia to address the concerns by providing stronger institutional support and establishing formal accreditation processes. Notably, it is a wider concern that PTs are receiving less recognition compared to other coaching professions (31, 32), though countries such as the United States and the United Kingdom possess the established accrediting bodies with standardised requirements (31, 32), while many Eastern European countries rely heavily on physical education university degrees or informal courses offered by either university institutions (37, 38) or informal providers (35, 36).

“*Being an effective PT*” storybook theme clearly illustrates a transformative journey among the interviewed PTs, transitioning from a theory-driven mindset to an understanding of common challenges and an appreciation for the diversity seen in human behavior, including clients' desires, competencies, as well as psychological and social dynamics. For example, one PT said that he would initially “*…approach all clients with similar expectations, assuming that if a particular method or routine worked for one person, it should work for everyone. However, after working with clients who struggled due to factors I had not initially considered—such as stress, mental health, or their lifestyle—it became clear that a one-size-fits-all approach does not work*.” Nearly all participants were in agreement about the significance of psychological aspects when working with people, which they came to recognise and value more and more as they gained experience. In the context of personal training, the power of relationship management must play a crucial role, as the effectiveness of emotional and social intelligence in facilitating happiness, confidence, and self-efficacy is essential to inspire and guide clients toward achieving their goals (73). Indeed, the importance of social charisma and positive messages has been promoted in many studies evaluating professional PTs’ work (30, 57, 59). For example, when 148 clients were asked to prioritise a PT with either exceptional knowledge of exercise sciences or exceptional social skills, the majority opted for the latter (30). Importantly, however, it should be stressed that a socially attractive gym environment could become a “trap” for those in need of behaviour change, leading them to prioritise comfort and enjoyment over the challenging steps necessary to positively impact metabolic health and wellbeing. One interesting comment from our investigation was, “*I have realised that the most successful transformations happen when clients are self-driven and motivated. While I can provide the tools and guidance, the real progress comes from clients who are willing to put in the effort consistently. In this respect his role is to build the motivation to commit to building [the client's] best selves…the drive has to come from within.”* Crucially, though, while the autonomous motivation and self-regulation skills may indeed be the best predictors of weight management results (74), research suggests that individuals who need obesity interventions require additional psychological and emotional support to modify their behaviour (27, 28), otherwise they may be less motivated to pursue it (27, 28, 75). This warns against passivity and provides a clear indication that a PT needs to be the one to initiate positive changes and inspire healthy behaviours both inside and outside of the gym for those in need.

The PTs from this study underlined the counselling duty of a PT who often had to take on the role of a psychologist who “*listens, motivates, and supports clients on a personal level*” and “*helps clients navigate emotional and lifestyle challenges to achieve lasting change”*. Furthermore, many pointed out to trust as a key factor enabling a PT to effectively influence their clients earned by “…*showing them that I genuinely care about helping them*.” This is a critical element in PT practice because a client's trust could provide a trainer with the opportunity to encourage them to make healthier decisions outside of the gym space (30, 58, 59). Therefore, it is plausible to suggest that the personal fitness training profession could hold significant potential for making a positive influence on public health (59) by promoting physical literacy (i.e., a positive relationship with being active) in adults who had not been previously inspired to engage in physical activity or learn about its importance (76). Physical literacy is a multidimensional concept suggesting in this context that PTs should aspire to help their clients have the confidence and competence to participate in various activities, understand the benefits of an active lifestyle for the entire life course, and help develop their motivation to maintain it (76). Hence, moving away from the emphasis on physical performance while finding the right time to deliver a strong message of support with exercise strategies may be the key for a successful practitioner. Moreover, teaching a client on “how” and “why” regarding the workout approaches could be indispensable for having them embarked on a continuous fitness journey. This was recognised by two PTs who underlined that “… *a significant measure of success is when clients feel confident enough to take control of their fitness and maintain healthy habits on their own*” and “…*(my) approach now includes not only physical fitness but also education around healthy habits and recovery, fostering a more sustainable and independent fitness journey for each client*.” Therefore, cultivating a strong sense of autonomy and understanding in clients is pivotal for long-term success, as equipping them with the knowledge and confidence to independently manage their fitness habits will promote sustained progress and commitment to a healthy lifestyle.

The primary limitation of this study was the absence of female PTs and their perspectives on working in the Serbian environment. Moreover, while the study aimed to capture insights from the most successful professionals in the field, the criteria were inherently subjective, relying on assessments from peers and managers of the gyms known for their exemplary work in specialised personal training services. This issue would suggest that an empirical study is needed to help define what it means to be an expert PT and to guide future investigations. The number of interviewed coaches (*n* = 12) could also be considered a relatively small sample albeit an informed and homogenous one for the purposes of this study. Resultingly, the findings provide valuable insights into the profession, offering a foundation for further research.

## 5 Conclusion and future directions

This study's aim was to gain the views of high-level PTs in order to learn more about their practice and developmental pathway in Serbia. Findings highlight the importance of evolving the personal training profession by shifting from a traditional, performance-focused approach to a holistic model that integrates psychosocial support and addresses the needs of a whole person. High-level PTs emphasised the value of methods and philosophies that prioritise client's wellbeing beyond physical fitness, underpinned by an education in psychology and self-directed learning. Given the complexity of implementing such a shift in a performance-driven industry, along with potential resistance from trainers, further exploration is needed to understand how this transition can be effectively achieved while balancing the evolving needs of clients and industry demands. Future investigations with a more diverse sample and a larger number of participants, including both trainers and clients, would help to build a more comprehensive understanding of what defines a PT in the context of health promotion and improvement. The findings from such investigations could help address the concept of physical literacy in adulthood which seems essential to be built upon. Another important insight from the study illustrates the gap in many PTs’ formal education related to biopsychosocial approaches, holistic frameworks, physical literacy, relationship management, and behavior change protocols, suggesting the potential for this research to shape industry standards, foster accreditation systems, or influence policy relating to all professionals that work to improve public health. These findings could provide critical guidelines to ensure that practitioners are equipped with the most relevant, evidence-based strategies to address evolving challenges and enhance overall effectiveness in their fields. To efficiently implement these recommendations, national bodies and accreditation organisations must play a role by establishing officially recognised courses that align with internationally accepted PT certifications. These bodies should set clear educational requirements, offer continuous professional development opportunities, and ensure that all training programmes conform to national accreditation standards. This would prevent the proliferation of unregulated courses and help establish a more consistent, high-quality educational framework for PTs, ultimately enhancing public health outcomes.

## Data Availability

The raw data supporting the conclusions of this article will be made available by the authors, without undue reservation.
